# SoundToxins: A Research and Monitoring Partnership for Harmful Phytoplankton in Washington State

**DOI:** 10.3390/toxins15030189

**Published:** 2023-03-02

**Authors:** Vera L. Trainer, Teri L. King

**Affiliations:** 1 Olympic Natural Resources Center, University of Washington, Forks, WA 98331, USA; 2 Washington Sea Grant, University of Washington, Shelton, WA 98584, USA

**Keywords:** amnesic shellfish poisoning, *Pseudo-nitzschia*, paralytic shellfish poisoning, *Alexandrium*, diarrhetic shellfish poisoning, *Dinophysis*, *Heterosigma*, *Protoceratium*, *Akashiwo*, *Phaeocystis*, SoundToxins

## Abstract

The more frequent occurrence of marine harmful algal blooms (HABs) and recent problems with newly-described toxins in Puget Sound have increased the risk for illness and have negatively impacted sustainable access to shellfish in Washington State. Marine toxins that affect safe shellfish harvest because of their impact on human health are the saxitoxins that cause paralytic shellfish poisoning (PSP), domoic acid that causes amnesic shellfish poisoning (ASP), diarrhetic shellfish toxins that cause diarrhetic shellfish poisoning (DSP) and the recent measurement of azaspiracids, known to cause azaspiracid poisoning (AZP), at low concentrations in Puget Sound shellfish. The flagellate, *Heterosigma akashiwo*, impacts the health and harvestability of aquacultured and wild salmon in Puget Sound. The more recently described flagellates that cause the illness or death of cultivated and wild shellfish, include *Protoceratium reticulatum*, known to produce yessotoxins, *Akashiwo sanguinea* and *Phaeocystis globosa*. This increased incidence of HABs, especially dinoflagellate HABs that are expected in increase with enhanced stratification linked to climate change, has necessitated the partnership of state regulatory programs with SoundToxins, the research, monitoring and early warning program for HABs in Puget Sound, that allows shellfish growers, Native tribes, environmental learning centers and citizens, to be the “eyes on the coast”. This partnership enables safe harvest of wholesome seafood for consumption in the region and helps to describe unusual events that impact the health of oceans, wildlife and humans.

## 1. Introduction

Washington State’s bounty of scenic beauty, natural resources and abundant marine waters is home to 8 million residents and is a destination for visitors from around the world. Native tribes have strong cultural connections with the sea, shellfish and fish in both Puget Sound and the Washington Coast, where for centuries, native people have lived by the precept, “when the tide is out, the table is set”. The social and cultural values of shellfish are ingrained in the livelihood of subsistence harvesters such as members of the Quinault Indian Nation, where the expression in their native language, “ta’aWshi xa’iits’os”, translates to “clam hungry”. This desire to maintain and increase access to shellfish has led many tribes to reestablish clam gardens for subsistence and ceremonial harvest and to establish shellfish farms for commercial production.

Shellfish growers farm more bivalve shellfish than any other state in the United States with the total farmed production valued at more than USD 150 million [1]. Aquacultured shellfish species include clams, oysters, mussels and geoduck. Washington has 115 commercial growing areas that represent harvestable shellfish acreage in either approved, conditional, restricted or prohibited status in which over 900 shellfish farms are permitted to operate [2]. The commercial production of shellfish occurs primarily in rural areas and provides critical family wage jobs. Some companies have been in operation for over 100 years, safely and sustainably producing shellfish for local, national and international markets.

Recreational harvesting on Washington’s beaches is a popular activity for both residents and visitors to the region, involving approximately 300,000 people [3], relying on clean water and shellfish free from toxins. In Washington, shellfish can be harvested from publicly owned tidelands. With over 350 public harvesting locations and about 275 shellfishing tides per year, there are bountiful opportunities to harvest shellfish on public lands. Puget Sound and coastal beaches are home to many varieties of bivalve shellfish, including manila clam (*Ruditapes philippinarum*), littleneck clam (*Leukoma staminea*), butter clam (*Saxidomus gigantea*), varnish clam (*Nuttalia obscurata*), macoma clam (*Macoma* spp.), horse clam (*Tresus capax* and *T. nuttallii*), eastern softshell clam (*Mya arenaria*), geoduck clam (*Panopea generosa*), razor clam (*Siliqua patula*), as well as mussels (*Mytilus trossulus* and *M. galloprovincialis)* and oysters (*Crassostrea* spp. *and Ostrea* spp.). Season openers vary by beach. Razor clams are only found on the outer coast because they require a high wave energy sandy beach to survive. The recreational razor clam harvest season usually occurs in fall through spring of each year. These abundant resources for subsistence, ceremonial, commercial and recreational harvest are threatened by both water quality downgrades and several species of naturally occurring algae. These algae can produce toxins of concern to human or animal health when environmental conditions, such as increasing temperature and changing salinity, encourage their populations to bloom. During these harmful algal blooms (HABs), the algae and their toxins are concentrated in the shellfish tissues as they feed on the bloom and transfer toxins up the food chain to wildlife and humans via direct ingestion of toxic shellfish or planktivorous fish. However, not all HABs impact human or marine mammal health. High concentrations of some algal species can cause bird, fish or shellfish mortalities in large numbers, either due to hypoxia or toxins that directly impact shellfish health, such as yessotoxins. Maintaining seafood security and shellfish health are essential to retain the cultural, social and economic benefits of shellfish to harvesters and consumers, thus the effective and timely monitoring of HABs and the toxins that they produce are critical.

### 1.1. Species of HABs in Puget Sound

There are many species of phytoplankton in Puget Sound that are of concern both for human consumption of shellfish and fish and shellfish health. One of the earliest identified and most deadly toxins is paralytic shellfish toxins (PSTs), produced by species of the dinoflagellate genus *Alexandrium*. The human illness syndrome, paralytic shellfish poisoning (PSP), is caused by the ingestion of shellfish or fish that contain these PSTs, resulting in symptoms such as tingling of the lips and tongue, short-term paralysis and death. Reports from Captain George Vancouver’s explorations of the region documented the death of one of his crew in 1793 after eating mussels contaminated with PSTs in the neighboring uncharted coastline of what is now known as British Columbia [4]. In the 1940s, the first three fatalities due to PSP were documented in Washington State shellfish [5]. Shellfish monitoring for PSTs by health officials was sporadic until the early 1970s, when levels in shellfish above the FDA regulatory limit of 80 μg/100 g shellfish tissue occurred in northern Puget Sound [5]. The Washington State Department of Health (WDOH) systematically monitors PSTs throughout the state’s marine waters, with most monitoring sites located in Puget Sound where most of the state’s recreational, commercial and subsistence shellfish harvest occurs. Since 1989, WDOH has deployed a sentinel mussel monitoring program [6] through which mussels are suspended from wire mesh cages below floats and docks. These mussels are sampled routinely and tested for PSTs using the mouse bioassay [7]. The high numbers of PST closures over time and space and the number of species of harvestable shellfish monitored by WDOH are summarized in [5], illustrating the need for phytoplankton monitoring as a complement to shellfish testing.

The marine diatom, *Pseudo-nitzschia*, can produce the toxin, domoic acid (DA), which when concentrated in shellfish tissues and consumed by humans, can cause amnesic shellfish poisoning (ASP). This human illness syndrome, ASP, can cause temporary or permanent short-term memory loss and even death, as was experienced by consumers of mussels from Prince Edward Island, Canada, during the first recorded ASP event in 1987 [8]. The first closures in the US due to ASP occurred on the outer coast of Washington State in 1991 [9,10,11], where razor clams were able to retain the toxin, DA, for up to 1 year [12]. In 1997, the WDOH reported the presence of DA at a shellfish farm in Puget Sound, but concentrations above the regulatory closure level of 20 ppm were not detected until 2005, impacting commercial and tribal shellfish harvest [13]. Since that year, DA closures in Puget Sound have been infrequent; however, costly closures of the razor clam and Dungeness crab (*Metacarcinus magister*) harvest occur almost annually on outer Washington coast beaches [14]. 

The dinoflagellates, *Dinophysis* and *Prorocentrum*, can produce the toxins, okadaic acid and dinophysistoxins, which cause diarrhetic shellfish poisoning (DSP) in humans (reviewed in [15]). Monitoring for diarrhetic shellfish toxins by WDOH began in 2011 when three people became ill with DSP at a campsite in northern Puget Sound after recreationally harvesting and consuming mussels. Elevated concentrations of several species of the marine dinoflagellate, *Dinophysis*, were observed by SoundToxins partners (see Section 1.2, below) at the time of this event, the first documented cases of DSP in the US. Levels of dinophysistoxins were measured in mussels at 2–10 times above the regulatory level of 16 μg/100 g [16].

In 2007, a research study that collaborated with SoundToxins documented the presence of several species of the small dinoflagellate, *Azadinium*, in Puget Sound [17], known to produce the toxins, azaspiracids, that cause a syndrome in humans called azaspiracid shellfish poisoning (AZP). The first cases of AZP were discovered after several people consumed cultivated shellfish from Ireland and suffered from symptoms similar to DSP, including nausea, vomiting, severe diarrhea and stomach cramps [18]. In Washington State, a new azaspiracid, named AZA-59 was identified [17], and levels of this toxin in shellfish measured well below the regulatory limit [19]. Although the risk of AZP is estimated to be low, continued vigilance in monitoring *Azadinium* will provide early warning of any increased risk.

Several species of marine flagellates documented by SoundToxins have contributed to summer shellfish mortalities in Puget Sound. Although these species have been present for centuries, they were largely unrecognized as agents impacting shellfish health because many other potential causes of ‘summer mortality’ were researched first and not identified as primary causes [20]. *Protoceratium reticulatum*, a producer of yessotoxins [21], has been measured in extremely high abundance in south Puget Sound, coincident with massive manila clam mortalities. In 2019, the highest concentration of *P. reticulatum* corresponded to 0.9 ug/L yessotoxin (YTX) in shellfish tissues [20], a concentration similar to those measured during massive abalone mortalites in South Africa [22] and clam mortalites in Chile [23]. Other flagellates, present in high concentrations in Puget Sound and known to kill shellfish in other areas of the world, are *Akashiwo sanginea*, believed to be responsible for mass mortalities in Texas, USA [24], China [25] and Peru [26] and *Phaeocystis globosa*, which has also been associated with Puget Sound shellfish mortalities [20].

*Heterosigma akashiwo* is a major killer of finfish, including cultivated Atlantic (*Salmo salar*) and Pacific (*Oncorhynchus* spp.) salmon [27] grown in marine net pens and in the wild. There are reports of wild salmon and marine fish mortality in Washington coastal waters [28,29]; however, “farmed” fish are particularly vulnerable because when winds or currents move the blooms into penned areas, the fish cannot escape the affected waters. *Heterosigma akashiwo* has caused the death of salmonids held in net pens in Puget Sound since at least 1976 [30,31] when the salmon aquaculture industry in Washington State suffered economic losses of ~USD 2 to 6 million per episode due to *H*. *akashiwo* blooms. More than 100 salmon hatcheries are operated in Washington State by the Washington State Department of Fish and Wildlife, Native American tribes, the U.S. Fish and Wildlife Service and private companies, such as Cooke Aquaculture and Long Live the Kings. The mechanism of *H. akashiwo* toxicity is not well understood; therefore monitoring of the presence of cells currently provides the best early warning of potential risk.

### 1.2. The SoundToxins Program

In 1998, the Olympic Region Harmful Algal Bloom (ORHAB) partnership was formed to better manage HABs on Washington’s rural outer coast. This collaborative partnership between the coastal tribes, researchers and state fisheries and health managers led to improved understanding of HABs, allowing refinement of selective shellfish harvest at safe beaches, resulting in fewer coastwide closures [32]. Formed in 2006, SoundToxins is modeled after the successful ORHAB program and adapted to address the HAB issues in Puget Sound. SoundToxins is the phytoplankton research and monitoring program for Puget Sound, created to provide an early warning to WDOH, aquaculture producers and tribal and state natural resource managers about the presence of potentially harmful plankton. SoundToxins benefits from inclusion of managers as full partners in the program, resulting in frequent communications to refine and improve data display, outreach and monitoring locations and frequency. The program aims to advance methods for the early detection of HABs and provide prediction of bloom events to protect humans from toxic shellfish, reduce shellfish recalls and lessen harvest losses. It is a diverse partnership consisting of native American tribes, shellfish farmers, fish farmers, environmental learning centers, nongovernmental organizations, universities and colleges, state natural resource and health managers and community members working side by side to monitor Puget Sound waters. It was conceived and initiated by the National Oceanic and Atmospheric Administration (NOAA) and is now directed by Washington Sea Grant (WSG).

**Figure 1:**
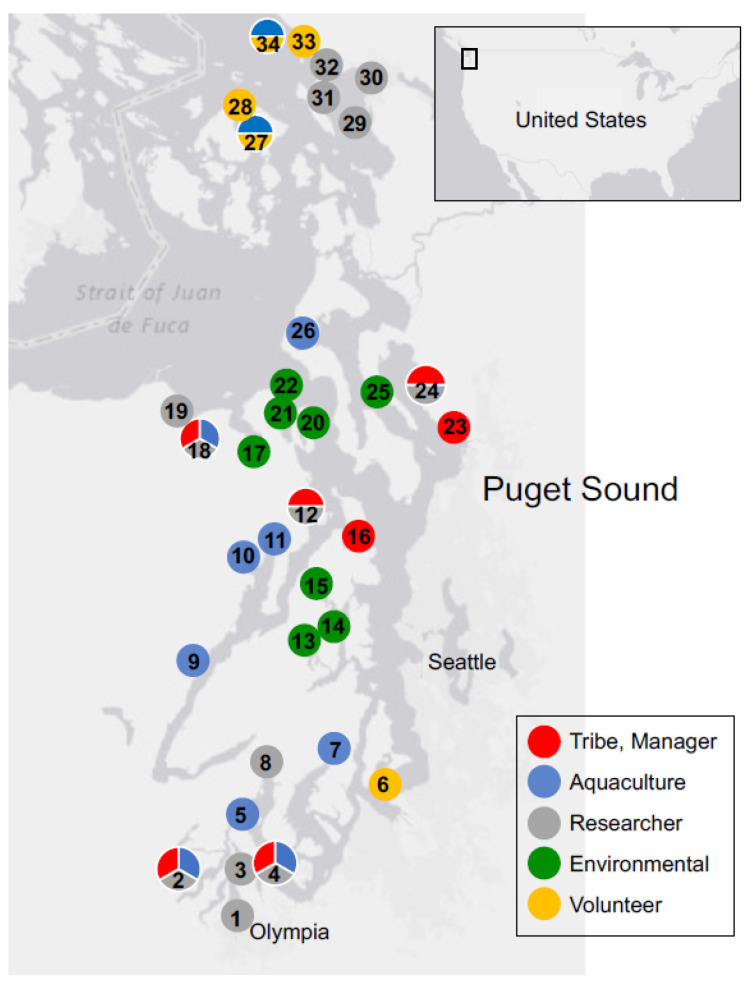
Numbered SoundToxins sampling sites. Partners are identified as Tribe and natural resource manager (red), aquaculturist (blue), research organization, including universities (gray), environmental learning center (green), and volunteer participants (orange). SoundToxins sites are: 1. Budd Inlet South, 2. Totten Inlet, 3. Budd Inlet North, 4. Nisqually Reach, 5. Spencer Cove, 6. Quartermaster Harbor, 7. Burley Lagoon, 8. North Bay, 9. Hama Hama, 10. Quilcene Bay, 11. Dabob Bay, 12. Hood Head, 13. Dyes Inlet, 14. Brownsville, 15. Liberty Bay, 16. Port Gamble, 17. Discovery Bay, 18. Sequim Bay South, 19. Sequim Bay North, 20. Mystery Bay, 21. Port Townsend, 22. Fort Worden, 23. Tulalip Bay, 24. Port Susan, 25. Camano Island, 26. Penn Cove, 27. Glenwood Springs, 28. East Sound, 29. Bellingham Bay buoy, 30. Fairhaven. 31. Gooseberry Point, 32. Birch Bay, 33. Blaine Fishing pier, and 34. Drayton Harbor. Two opportunistic sites in Willapa Bay on the outer coast of Washington are not shown. All tribes are co-managers of marine resources within their usual and accustomed areas and sole managers of reservation lands. Management partners not shown include WDOH, Washington Department of Fish and Wildlife and NANOOS. WSG is the current director of SoundToxins.

SoundToxins has grown from 4 partners in 2006 to more than 30 partner organizations in 2022 (Figure 1). On the outer coast, shellfish companies collaborate with SoundToxins, and two farms are participating in a NOAA-funded project to better understand shellfish-killing HABs that impact aquaculture in Willapa Bay. The program partners are well-trained, and many are paid by their companies, tribes, universities, organizations, or agencies to conduct the monitoring, while others volunteer their time out of a commitment to better understand their local Puget Sound stretch of coastline. Often, volunteers join SoundToxins because they have previous careers in the sciences, such as veterinary or biomedical science. The three primary goals of SoundToxins are to: 1. document unusual bloom events and new species entering Puget Sound, 2. provide alerts on increasing harmful phytoplankton concentrations, and 3. determine the environmental factors that promote the onset and flourishing of HABs.

## 2. Results

**Figure 2:**
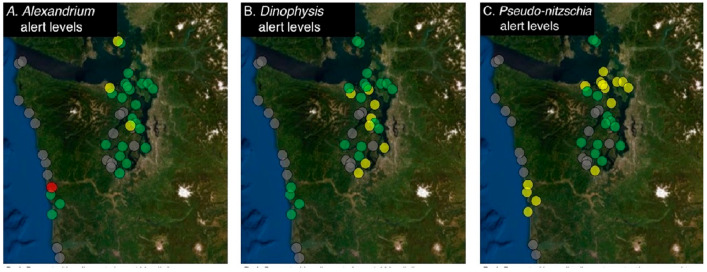
Example map for the reportable HAB genera *Alexandrium, Dinophysis* and *Pseudo-nitzschia*. Symbols correspond to the following cell threshold levels for: (**A**) *Alexandrium*, abundance above 100 cells/L (red), abundance between 1–99 cells/L (yellow), absent (green); (**B**) *Dinophysis*, abundance above 1000 cells/L (red), abundance between 1–999 cells/L (yellow), absent (green); (**C**) *Pseudo-nitzschia*, threshold levels were established to distinguish the risk between small and large cell type: small cell count greater than or equal to 1,000,000 cells/L or large cell count greater than or equal to 50,000 cells/L (red), small cell count below 1,000,000 cells/L and large cell count below 50,000 cells/L (yellow), absent (green). A gray dot indicates that data have not been entered for that site within the past 2 weeks. Required reporting for shellfish-killing HABs has recently been added to SoundToxins, and those maps are not shown here.

Since 2011, the SoundToxins online database allows WDOH, other natural resource managers and participants to view near-real time data on maps developed for each species of concern (see Figure 2). When the program was established in 2006, the data required individual interpretations of cell abundances to assess risk, and maps were not available in real time. In 2011, with the first occurrence of DSP in Puget Sound, WDOH requested that SoundToxins create a system that would allow the risk of HABs to be assessed as a summary map, without having to study the database (Figure 2). This led to the development of separate risk maps for *Alexandrium*, *Pseudo-nitzschia* and *Dinophysis* and was expanded to maps of additional reportable species in 2022. The data entered by SoundToxins partners and the resulting maps are reviewed daily by WSG staff for quality and accuracy.

**Figure 3:**
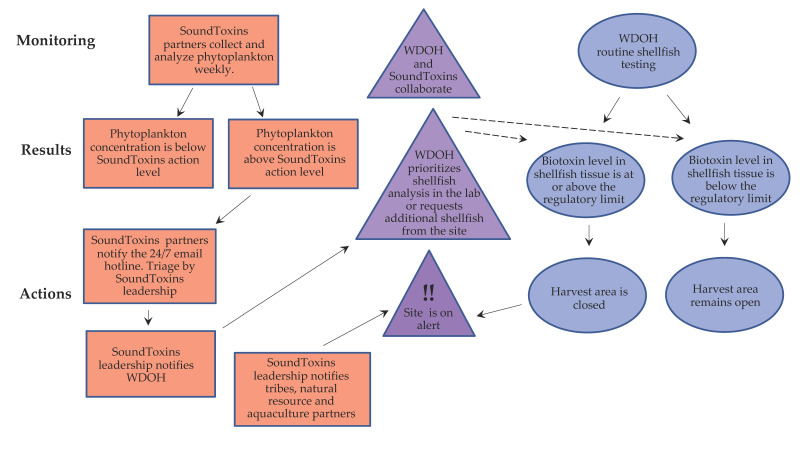
Integration of SoundToxins phytoplankton monitoring with the WDOH shellfish biotoxin program. One of the key elements of the SoundToxins collaboration is assisting with prioritization of samples for toxin analysis. A greater number of shellfish samples arrive at WDOH than can be analyzed in one day; therefore; a strategic approach involves prioritizing shellfish testing on samples where *Alexandrium, Dinophysis* or *Pseudo-nitzschia* are observed. Areas are reopened for harvest using WDOH shellfish biotoxin data and cannot be reopened using SoundToxins data alone. Sample triage by SoundToxins leadership includes confirmation of HAB species by microscopy and ensuring that HAB alert levels are correctly assigned. Solid arrows show the natural flow of information; the dotted arrows represent WDOH management decisions made possible through collaboration with SoundToxins. Red boxes show SoundToxins activities, blue ovals show WDOH activities and purple triangles represent collaborative activities. Databases used for entering SoundToxins data are accessed through the SoundToxins website [33], and WDOH shellfish toxin data are used to create Shellfish Safety Maps [34]. SoundToxins leadership alerts WDOH by e-mail and then phone if the email is not acknowledged. WDOH alerts shellfish growers and tribes in the impacted area by phone first, then by e-mail, and finally by notating biotoxin closures on the Commercial WDOH Shellfish Safety map. Recreational shellfish harvesters are alerted by the Recreational Shellfish Safety Map, a toll-free hotline and by signage posted at harvest sites.

Communications by SoundToxins to WDOH Marine Biotoxin staff include e-mails and phone calls when the various threshold levels of *Alexandrium, Pseudo-nitzschia* and *Dinophysis* have been observed. Similarly, data are entered into the SoundToxins database as soon as possible after samples have been analyzed. The interactive relationship between the WDOH shellfish monitoring program and SoundToxins is shown in Figure 3. The number of alerts provided by SoundToxins to the WDOH over the last 6 years is shown in Table 1. Potential actions that are triggered by SoundToxins alerts to WDOH include prioritization of shellfish samples from at risk sites, collection of additional samples or pre-emptive closure of shellfish harvesting areas.

SoundToxins monitoring and rapid communication have resulted in successes that demonstrate the program’s effectiveness in protecting public health. For example, WSG and partners were recently awarded a NOAA grant to document the social and economic benefits of the SoundToxins program. Here, we highlight two of many success stories that have resulted from the SoundToxins partnership. Our first story occurred in the summer of 2011 when three people who had gathered and eaten shellfish at a campsite in north Puget Sound were poisoned. During this DSP incident, SoundToxins partners from the Jamestown S’Klallam Tribe alerted WDOH to elevated levels of *Dinophysis* spp. As a result, WDOH began including diarrhetic shellfish toxins as part of its routine testing in 2012. The occurrence of DSP in northern Puget Sound led to the development and funding of a research proposal to NOAA to study lipophilic toxins, including dinophysistoxins, yessotoxins and azaspiracids. This research involved SoundToxins partners who conducted the field sampling. This yielded research studies that have described the presence of high concentrations of yessotoxins (YTX; [16,20]) and several species of the dinoflagellate, *Azadinium* [17], at locations throughout Puget Sound.

In the second SoundToxins success story which occurred in spring 2015, a SoundToxins partner at the Taylor Shellfish hatchery noted high numbers of *Alexandrium* in a water sample collected from Dabob Bay (see Figure 1 for location) and immediately notified SoundToxins and WDOH. A shellfish sample was immediately requested by WDOH for analysis. When the toxin content of this sample was determined to exceed the safe harvest level, the commercial and recreational shellfish growing area in Dabob Bay was immediately closed, and other growing areas nearby were placed on alert. Multiple trucks bound for retail and wholesale markets, carrying thousands of pounds of potentially PSP toxic mussels, were halted. Without the identification and alert of *Alexandrium* by a SoundToxins partner, these shellfish would not have been tested by WDOH because that date fell outside the scheduled biotoxin testing period at this site. The early warning provided by SoundToxins very likely averted human illness and costly recalls from sales of toxic shellfish.

## 3. Discussion

The goal of the WDOH Marine Biotoxin Program (MBP) is to protect humans from illness and death caused by eating shellfish contaminated with biotoxins. The program monitors commercially and recreationally harvested molluscan shellfish, including clams, mussels, oysters, geoduck and scallops. In general, testing of shellfish for marine biotoxins by the WDOH occurs at 2-week intervals; however, some areas are monitored less frequently [6]. The SoundToxins partnership was established to provide routine phytoplankton data to alert the WDOH of HABs that could result in shellfish toxicity, especially during these interim periods when WDOH is not scheduled to monitor. SoundToxins sites were strategically established with WDOH input to focus on locations where HABs have historically occurred, where key shellfish resources are located or where additional sampling is needed. When SoundToxins documents a high risk of HABs, the number and variety of shellfish tested by WDOH often increases and is prioritized for that site (Figure 3).

During the first few weeks of the COVID-19 pandemic in Washington State in 2020, the work of SoundToxins was deemed essential by the WDOH. An urgent request to continue the program was received from the State of Washington by SoundToxins to provide critical information that allowed commercial and recreational shellfish harvesting to safely continue. The WDOH relied more heavily on the SoundToxins partnership because its staff was re-assigned to assist with COVID-19 testing, leaving them with severely reduced capacity for shellfish testing during the peak biotoxin season, from May–October, when the lab often analyzes more than 70 shellfish samples per day. The 27 March 2020 letter which was sent by WDOH to WSG stated, “The need for early detection of HABs by our SoundToxins partners has become an increasingly critical component of the MBP. The vast volunteer network and expertise of SoundToxins leadership greatly enhances our ability to prioritize biotoxin samples while the Public Health Laboratory runs at near minimum capacity for testing shellfish samples for marine biotoxins. As we near the peak biotoxins season, state, local and tribal partners are increasingly focused on managing direct community threats from COVID-19. Having the SoundToxins early warning system component in place ensures that we and our partners collect and test regulatory samples when and where they pose an imminent threat to human health”. Furthermore, MBP could not receive samples from all areas during COVID-19 due to stay-at-home orders; therefore, some shellfish harvest locations were closed using SoundToxins data alone.

### 3.1. Evolution of the SoundToxins Partnership

The SoundToxins partnership has evolved since its inception in 2006. A series of interviews with SoundToxins participants provided key recommendations to strengthen the program [35]. The recommendations included providing routine communications with partners by documenting how data were being used and summarizing recent HAB events. The needs for an institutional arrangement and for a volunteer coordinator to work with participants, manage data and streamline communications with the WDOH were also documented. As a result of this study, WSG joined the SoundToxins program to provide day-to-day management of the program.

### 3.2. Research and Monitoring Benefiting from SoundToxins

SoundToxins has provided a guidebook for the establishment of phytoplankton monitoring for HAB early warning [36] that has been used in other geographical locations. The Southeast Alaska Tribal Ocean Research Partnership [37] was developed using SoundToxins methods and advice [38] by a former SoundToxins participant who moved to Alaska. Alaska and Oregon phytoplankton data and ORHAB data are entered into the SoundToxins database with the goal to unify the HAB early warning system on the U.S West Coast. Similarly, the SoundToxins methods have been shared with researchers in neighboring British Columbia, Canada [39] and other locations around the world. 

Several research projects have been developed with SoundToxins (Table 2), forming the cornerstone for monitoring, essential for providing data, phytoplankton cultures and a framework for collection of additional samples needed for these investigations. These projects have involved teams of national and international investigators who have collaborated with SoundToxins colleagues to rely on local knowledge to help solve worldwide HAB problems. Graduate student projects (three Master’s and two Ph.D.) have been made possible through collaboration with SoundToxins. Topics range from the description of environmental regulators of new HAB risks, the characterization of new toxins and the incorporation of emerging new tools and technologies for the optimal observation of environmental change in Puget Sound. In addition to the projects listed in Table 2, SoundToxins has contributed to annual Puget Sound Ecosystem Monitoring Program publications, with the goal to collaborate across monitoring programs in the region [40].

### 3.3. Funding the SoundToxins Program

SoundToxins was originally supported through NOAA’s Oceans and Human Health Initiative with funding from the West Coast Center for Oceans and Human Health. Congress zeroed out NOAA’s budget for this program in 2012, leaving a significant funding gap. Washington Sea Grant, now the lead organization for SoundToxins, joined as a program partner in 2008 and assumed the tasks of volunteer coordination, data management and communications. This management of SoundToxins has been sustained through external research grant funding from sources such as the Washington State Department of Ecology, NOAA competitive research grants and the Northwest Association of Networked Ocean Observing Systems (NANOOS), all of which are limited in their ability to provide funds for an ongoing monitoring program. A 2011 study recommended that a secure source of funding should be found for SoundToxins [35]. In fact, HAB monitoring has been supported on the outer coast of Washington (for ORHAB), which could be used as a model for acquiring sustained support of SoundToxins. While ORHAB is funded by Washington House Bill 1620, which includes a surcharge of USD 2–4 on licenses for shellfish harvesting, both in Puget Sound and along the outer coast, funding for SoundToxins is not included. However, a study published in 2014 showed that shellfish harvesters were willing to pay an average of USD 5 more for their annual shellfish license if this would help reduce the number of beach closures due to HABs [63], so an additional tax might be considered to provide sustained support SoundToxins in the future.

### 3.4. The Future of HAB Monitoring

Recent studies have documented the transfer of freshwater toxins into the marine environment and bioaccumulation of low levels in Puget Sound shellfish [64]. The appearance of these freshwater toxins, such as microcystins, at marine shellfish growing areas near rivers and in freshwater lakes near Puget Sound [3] illustrate the importance of adaptation of marine monitoring programs to include new toxins [3]. The threat of these combinations of marine and freshwater toxins in shellfish is poorly understood, and the risk of freshwater toxins in marine shellfish is often not quantified. Therefore, the additive or synergistic toxicity of co-occurring blooms in lakes and marine waters must be studied to establish appropriate threshold levels for the early warning of combined HAB impacts at the marine and freshwater interface, a potential area of future research in which SoundToxins could play a larger role.

SoundToxins provides a unique framework for expansion of monitoring from a focus on HABs to a more comprehensive program that monitors for multiple stressors that will fluctuate with climate change, such as marine heatwaves, ocean acidification and low oxygen events. Cyanobacteria and some marine HABs favor warm temperatures and other environmental conditions, such as increased nutrient inputs from land, that will be associated with climate change [65]. Long-term climate projections for the Pacific Northwest suggest that rain events will increase. These may influence nutrient runoff from impervious surfaces, particularly as land is developed and regional populations increase [66]. In Puget Sound, the annual rainfall is projected to increase by up to 30% in the 2050s relative to the 1970–1999 baseline [67]; together with increases in temperature, this will result in enhanced stratification in the oceanic photic zone. Most of the extreme marine HAB events around the world are due to dinoflagellates (e.g., [68]) that have a competitive advantage over non-flagellated phytoplankton under stratified conditions, expected to increase with climate change, especially in the northeast Pacific [69]. These combined factors will promote a more favorable environment for both the marine dinoflagellate HABs [70] and freshwater cyanobacterial HABs, increasing the risk of toxic events that will threaten shellfish and fish health and safety for human consumption. 

Partnerships such as SoundToxins provide protection against these combined pressures that threaten the lucrative shellfish and fish resources of Puget Sound. In the future, SoundToxins will continue to provide early warning that will lead to adaptive strategies, such as early harvest, enhanced filtration at aquaculture facilities or changed location of shellstock, allowing growers and harvesters to continue to benefit from the bountiful marine resources of Puget Sound. SoundToxins is a dynamic partnership whose diversity, energy and quest for information is the driving force for its excellence. It is exemplary in its demonstration that collaborative, inclusive and committed programs succeed by collaborating toward common goals. SoundToxins prides itself on its iterative process that optimizes its protocols, outreach and communication strategies, noting that this monitoring and research program will continue to benefit the Puget Sound region and beyond well into the future. 

## 4. Materials and Methods

SoundToxins partners participate in an intensive group training workshop, onsite individual training and refresher courses throughout the year. Each partner receives a manual with detailed methods [36] that ensures consistent techniques and protocols are used throughout Washington State for HAB species monitoring. The methods listed below are described in more detail in the SoundToxins manual and Quality Assurance Project Plan (QAPP), which was established as part of the Puget Sound National Estuary Program. This document established measurement quality precision standards, including acceptable relative standard deviations of measurements and expected ranges of results. The plan included sample location and frequency, field measurement types, statistical analysis and detailed sampling protocols, including equipment decontamination, sample labeling and field log requirements.

Program partners sign an agreement that includes their commitment to the following elements: 1. collect samples weekly from the assigned sampling site(s), 2. analyze phytoplankton microscopically, providing cell counts for required species and relative abundance determinations for the remaining species within 48 h of sample collection, 3. send an e-mail message to the SoundToxins coordinator (soundtox@uw.edu) if alert levels for reportable species have been reached or exceeded, 4. enter phytoplankton and environmental data into the SoundToxins database within 3 days of sample collection, 5. attend the annual SoundToxins meeting, and 6. maintain the commitment to diversity, equity and inclusion. Participants that do not monitor as part of their paid work (i.e., tribal biologists and shellfish producers) are volunteers with the University of Washington (i.e., community members), providing them with insurance during their SoundToxins activities. Some partners have participated on West Coast NOAA cruises conducting critical phytoplankton research. Many have also completed the US Harmful Algae Training Program at Bigelow Laboratory in Maine. Beyond monitoring, participants are encouraged to conduct outreach and other activities to educate their community about phytoplankton. Some participants provide regular newspaper and social media reports in their communities; others have hosted phytoplankton art installations or have given seminars in classrooms and community centers and have conducted dockside phytoplankton sampling demonstrations.

### 4.1. Phytoplankton Collection and Identification

Samples are collected weekly from March to October and every other week in the winter. Current SoundToxins monitoring sites are shown in Figure 1.

#### 4.1.1. Whole Water Sample

A whole water sample is collected in a 2 L Nalgene bottle by placing it below the surface of the water or by using a bucket, then filling the bottle. This sample is then transported to the lab for processing and analysis. An aliquot of the live whole water sample is observed under the microscope to confirm that *Heterosigma* is present based on its swimming behavior. Then, abundance is determined using the whole water sample preserved with 1% formalin (final concentration). At selected sites, chlorophyll, nutrient, particulate and dissolved toxin samples are obtained from the whole water sample and submitted to WSG for analysis at the University of Washington Marine Chemistry Laboratory or the Northwest Indian College in Bellingham, WA, USA. 

#### 4.1.2. Concentrated (10×) Whole Water Sample

The 2 L Nalgene bottle whole water sample is inverted to mix, and a 50 mL aliquot is poured into a 50 mL glass test tube. Buffered formalin fixative (1 mL) is added to a final concentration of 1%, the tube capped and inverted to mix. After 24 h or more of settling, the upper 45 mL of seawater is removed using a pipette without disturbing the settled sample. The remaining 5 mL of the sample is transferred to a 20 mL scintillation vial. The 10× sample is used to count *Pseudo-nitzschia* using the Palmer–Maloney slide.

#### 4.1.3. Vertical Net Tow

A weighted 0.2 µm-mesh phytoplankton net (Eastside Research Nets, Redmond, WA, USA) is dropped from a dock or pier to 1 m from the bottom. The net is pulled to the surface at a constant speed (about 1 m/s), and the distance towed is noted by counting the meter marks on the net tow line. The net is pulled through the water column up to three times. If color is seen after the first or second pull, sampling is stopped. The concentrated plankton sample in the cod end is mixed gently before pouring it into a collection bottle. In the lab, the collection bottle is swirled gently, and a subsample is placed in a 20 mL scintillation vial containing 1 mL buffered formalin fixative. The net tow sample is used to count *Alexandrium, Dinophysis, Protoceratium, Phaeocystis* and *Akashiwo*.

#### 4.1.4. Microscopy

A 0.1 mL net tow or whole water sample is pipetted into a Palmer–Maloney cell and quantified microscopically (Motic BA31E Phase Contrast Microscope, Barcelona, Spain). All cells are enumerated at 200× magnification using a Palmer–Maloney counting slide (Electron Microscopy Sciences, Hatfield, PA, USA). The total volume within the Palmer–Maloney counting chamber is 0.1 mL, which must be taken into account when calculating the final concentration in cells/L.

### 4.2. Environmental Monitoring

Air and water temperature measurements, wind speed, tidal height and salinity are measured at the monitoring sites when water samples are collected. Air and water temperature are measured using a thermometer, and salinity is measured using a refractometer (Fisher Scientific; Hampton, NH, USA). Local tide charts are used to estimate tidal height. Wind speed is estimated using the Beaufort scale [71].

### 4.3. Phytoplankton Enumeration

Detailed methods used for determination of phytoplankton abundance can be found in the SoundToxins manual [36]. Abundance of *Pseudo-nitzschia* is determined from the 10× concentrated whole water as the majority of *Pseudo-nitzschia* are found near the surface. Abundances of *Dinophysis, Alexandrium, Heterosigma, Phaeocystis, Protoceratium* and *Akashiwo* are determined from the vertical net tow sample to capture these dinoflagellates that are often found in subsurface layers. All counts are performed using Palmer–Maloney counting chambers. Cell abundances are recorded in the SoundToxins database as cells/L. 

### 4.4. Phytoplankton Alert Levels

The alert levels for HAB species were determined using data from past SoundToxins and ORHAB monitoring and are intended to provide the maximum possible early warning to health and natural resource managers, tribes and aquaculture producers. SoundToxins partners immediately report the following to soundtox@uw.edu: any observation of *Alexandrium,* the first occurrence of *Dinophysis* and any subsequent increases in abundance from one week to the next. SoundToxins is alerted to *Pseudo-nitzchia* using the following guidance: if the large-celled *Pseudo-nitzschia* species, including *Pseudo-nitzschia australis, P. heimii, P. fraudulenta, P. pungens* and *P. multiseries* exceed 50,000 cells/L or if the small cells (*P. pseudodelicatissima, P. delicatissima* and *P. cuspidata*) exceed 1 million cells/L (see [32] for justification for these thresholds), these observations are reported to soundtox@uw.edu and recorded in the database. The threshold levels corresponding to colored dots on maps of reportable HAB species are converted to “traffic light patterns” (see Section 4.7, below, for details) shown in Figure 2.

For the potential shellfish-killing phytoplankton, *Protoceratium, Akashiwo* and *Phaoecystis*, alert levels are: when cells are first observed (yellow) and when cell counts are above 1000 cells/L (red). Any presence of *Heterosigma* is reported. For all reportable species, a sampling site is shown as a gray dot if no data are reported for more than 14 days (April–October) or not sampled for more than 30 days (November–March). A summary of thresholds for abundance (cell count action levels) used to alert shellfish managers is shown in Table 3.

### 4.5. Communications

Phytoplankton that threaten human, fish and shellfish health trigger alerts (an immediate email to soundtox@uw.edu) to the SoundToxin program coordinator (see above for designated alert levels). The reportable algal species involved in harmful human health risks are *Pseudo-nitzschia*, *Alexandrium and Dinophysis.* The reportable algal species having potentially harmful effects on fish and shellfish are *Heterosigma*, *Protoceratium*, *Akashiwo* and *Phaeocystis*. Partners also record what species is at highest abundance (blooming) for each sampling date and location. SoundToxins partners are encouraged to send alerts, questions and photos to the SoundToxins email hotline that is monitored 24 h a day 7 days a week. All alerts reported to soundtox@uw.edu are verified by SoundToxins leadership, and WDOH, tribes, natural resource, or aquaculture partners are subsequently notified, depending on the HAB species of concern. Monitoring summaries are sent at least monthly to all participants through a listserv that also provides additional educational opportunities for participants. SoundToxins staff also hold ‘office hours’ every few weeks for partners to gather virtually to discuss topics of interest, identification challenges and recent accomplishments. The communication strategy among SoundToxins partners, leadership and WDOH is shown in Figure 3.

### 4.6. Data Entry

Data are entered into the SoundToxins database [72] within three days of sample collection. Most enter data within 26 h of sampling. The SoundToxins database allows users to view/enter sampling events and species data. Each sampling event is considered a visit for which date/time, water temperature, tidal height, salinity and associated phytoplankton data are recorded. Each visit is associated with multiple phytoplankton genera/species observations (cell count, relative abundance, etc.). The required reportable species must have either cell counts or “none” reported.

### 4.7. Mapping

Data are entered into an online database that generates maps with threshold levels that are converted to green, yellow or red dots at each sampling site as appropriate. Maps are created in Oracle APEX (Redwood City, CA, USA) using the Map Region feature and Oracle Spatial. Dots are gray if data have not been entered within the past 2 weeks. Data can be accessed in real time by natural resource managers to make timely decisions about which harvest sites need additional samples or where shellfish should be harvested in advance of closures.

## Figures and Tables

**Table 1:** Number of alerts provided annually by SoundToxins to WDOH.

Year	Alert to WDOH
2022	43
2021	42
2020	62
2019	56
2018	34
2017	59

**Table 2:** Refereed publications that acknowledge or highlight collaboration with SoundToxins.

Year	Reference	SoundToxins Contribution	Publication Description	Lead Institution(s)
2023	[41]	sample collection, sharing of cultures	Characterization of the toxicity of a new azaspiracid, AZA-59	Alfred Wegener Institute, Germany
2022	[42]	sample collection, sharing of cultures	Comparison of *Dinophysis* species and their toxicity in the US	Virginia Institute of Marine Science
2022	[43]	methods sharing	Global HAB monitoring networks	University of Alabama
2021	[44]	data sharing	United States HAB trends	Woods Hole Oceanographic Institution
2021	[45]	data sharing	Global HAB trends	University of Tasmania
2021	[20]	data sharing, emergency response	Shellfish killing HABs	Washington Sea Grant
2020	[46]	methods sharing	Establishment of Alaska HAB monitoring program using SoundToxins methods	University of Alaska
2019	[47]	methods sharing	Scaling up from regional case studies to a global HAB observing system	Southern California Coastal Observing System
2019	[48]	data sharing, additional sampling	Characterize a possible new HAB threat, *Azadinium*	Second Institute of Oceanography, China; NOAA
2019	[49]	data sharing, additional sampling, lab analyses	Characterization of *Dinophysis* in south Puget Sound	The Evergreen State College (Master’s thesis)
2017	[50]	additional sampling	Genetic characterization of *Pseudo-nitzschia*	NOAA (Master’s thesis)
2017	[17]	additional sampling, data sharing	Characterize possible new HAB threat, *Azadinium*	Hanyang University, Korea (Ph.D. thesis)
2017	[51]	provide samples for culturing	Characterize optimal growth conditions for *Alexandrium*	NOAA
2016	[52]	provide samples for culturing, additional sampling, data sharing	Characterize optimal growth conditions for *Heterosigma*	San Francisco State University
2017	[53]	additional sampling, data sharing	Seagrass as a natural control mechanism for HABs	Hokkaido Univ., Japan; NOAA (Ph.D. thesis)
2015	[3]	data and methods sharing	Comparison of marine and freshwater HAB sampling in Washington State	NOAA, DOH
2015	[54]	additional sampling for *Vibrio*	Identification of environmental influences on *Vibrio* occurrence	NOAA
2014	[55]	data sharing	Seasonal variation of the genus *Dinophysis* within Puget Sound, Washington	Evergreen State College (Master’s thesis)
2014	[56]	additional sampling, data sharing	Defining optimal growth conditions for *Heterosigma akashiwo*	San Francisco State University
2013	[39]	method sharing	Establishment of British Columbia, Canada, phytoplankton monitoring networks	British Columbia Center for Disease Control
2013	[16]	additional sampling, data sharing	Describing the first diarrhetic shellfish poisoning event in the USA	NOAA
2013	[57]	additional sampling	Analysis of rapid toxin analysis methods for diarrhetic shellfish toxins	NOAA
2013	[58]	additional sampling for *Vibrio*	Development of a method for *Vibrio* detection	University of Washington
2011	[35]	methods sharing, interviews	Analysis of the SoundToxins partnership	University of Washington (Master’s Thesis)
2010	[59]	methods sharing	Description of SoundToxins partnership	University of Washington
2009	[60]	data sharing	Characterization of paralytic shellfish toxin trends in Puget Sound	NOAA
2008	[61]	additional sampling, data sharing	Characterization of saxitoxin in Puget Sound *Alexandrium*	NOAA
2007	[13]	additional sampling, data sharing	Characterization of domoic acid closures in Puget Sound	NOAA
2006	[62]	additional sampling, data sharing	Characterization of domoic acid closures in Puget Sound	NOAA

**Table 3:** Phytoplankton abundance and toxicity reportable by SoundToxins to shellfish managers.

Marine Toxin	Known Causative Organism(s) in WA	Regulatory Method	Shellfish Toxin Regulatory Level (WDOH)	# Shellfish ≥ Regulatory Level in Puget Sound (# Samples Tested) in 2012	Maximum Concentration in Shellfish 2012	Water or Particulate Toxin Action Level	Cell Count Action Level	Year of First Known Illness or Mortality in WA State
Saxitoxins	*Alexandrium*	Mouse bioassay	80 µg/100g	59 (2101)	10,304 µg/100g	~100-200 ng STX equiv./L	*Alexandrium* present	1942
Domoic acid	*Pseudo-nitzschia*	HPLC	20 ppm	0 (1305)	7 ppm	~200 ng/L	>50,000 cells/L (large *Pseudo-nitzschia*) and >1,000,000 cells/L (small *Pseudo-nitzschia*)	1991
Diarrhetic shellfish toxins	*Dinophysis*	LC/MS-MS	16 µg/g	87 (903)	184 µg/g	~20 ng/L	>20,000 cells/L	2011
Yessotoxin	*Protoceratium reticulatum*	n/a	3.75 mg/kg ^a^	n/a	n/a	~200 ng/L	>1000 cells/L	2018
Toxin not identified	*Akashiwo sanguinea*	n/a	n/a	n/a	n/a	n/a	>1000 cells/L	n/a
Toxin not identified	*Phaeocystis globosa*	n/a	n/a	n/a	n/a	n/a	>1000 cells/L	n/a

^a^ EU regulatory action levels; yessotoxin is not regulated in the USA. WDOH = Washington State Department of Health; LC/MS-MS = liquid chromatography tandem spectrometry; STX = saxitoxin; HPLC = high performance liquid chromatography; ppm = parts per million; # = number; n/a = not applicable. Modified from [3].

## Data Availability

No new data were created or analyzed in this study. Data sharing is not applicable to this article.

## Dedication

To Rita Horner whose knowledge, guidance and support for SoundToxins have formed the cornerstone of our partnership and whose book has been an irreplaceable resource for the many phytoplankton enthusiasts in the Pacific Northwest.
